# A Comparative Analysis of Clinical Features of Diabetes Mellitus Type 2 With Respect to Duration of Diabetes

**DOI:** 10.7759/cureus.74849

**Published:** 2024-11-30

**Authors:** Jai Kumar, Talha Rehman, Rifa Barkat, Bibi Laraib Shah, Lareb Sindhu, Muhammad Husnain, Mohsin Jamil Siddiqui, Atif A Hashmi

**Affiliations:** 1 Internal Medicine, Hamdard College of Medicine and Dentistry, Karachi, PAK; 2 Internal Medicine, Ziauddin University, Karachi, PAK; 3 Family Medicine, Sindh Healthcare Commission, Karachi, PAK; 4 Internal Medicine, Anabiya General Hospital, Karachi, PAK; 5 Pathology, Liaquat National Hospital and Medical College, Karachi, PAK

**Keywords:** diabetes mellitus, duration of diabetes mellitus, duration of symptoms, symptoms, type 2 diabetes symptoms, types 2 diabetes

## Abstract

Objectives

Diabetes mellitus type 2 is a chronic metabolic disorder characterized by insulin resistance and progressive beta-cell dysfunction. As diabetes persists over time, more pronounced symptoms like polyuria, polydipsia, fatigue, and complications like neuropathy, retinopathy, and cardiovascular issues may develop. Therefore, this study assessed the clinical symptoms associated with type 2 diabetes regarding the duration of diabetes.

Methodology

This cross-sectional study was conducted in a secondary care hospital, using a non-probability convenient sampling method. Patients visiting the outpatient clinics were recruited in the study after obtaining informed written consent from them. The duration of the study was about six months from March 1, 2024, to August 31, 2024. A sample of 450 type 2 diabetic patients, aged 40-65 years, was included in this study. The study identified patients with type 2 diabetes by using their altered glycosylated hemoglobin (HbA1c) level recorded within the last 30 days, which reflects glycemic control. The demographic information, including age, gender, socioeconomic status, health condition, co-existing illnesses, and diabetes-related symptoms was also collected from the patients. SPSS software was used for data analysis. A chi-square and one-way analysis of variance (ANOVA) were employed to determine the association of clinical symptoms in type 2 diabetes mellitus.

Results

The study findings showed that the mean age was significantly higher in patients with diabetes for less than one year (60.44±15.88 years) compared to those with diabetes for one to five years (52.26±14.37 years) and more than five years (53.95±14.28 years) (p<0.001). The association of gender, comorbidities, and socioeconomic status with the duration of diabetes revealed a significant difference (p<0.001). Frequent urination was significantly more common in the less than one-year group 89(59.3%) compared to the one to five years 54(36.0%) and more than five years 11(7.3%) (p<0.001).

Conclusion

Diabetes mellitus is a very prevalent systemic disease, with various musculoskeletal and psychosocial symptoms. The frequency of these symptoms varies with respect to the duration of diabetes. In this study, we outlined the various symptoms of diabetes and their relation to the duration of diabetes. Among various symptoms, dyspnea, chest tightness, and muscle cramps increase with increasing duration of diabetes.

## Introduction

Diabetes refers to a group of diseases described by elevated blood glucose levels. It occurs owing to a deficiency in the production or function of insulin, or both, which can be due to numerous causes, leading to metabolic disorders of proteins and lipids [1]. Over time, hyperglycemia can result in tissue and organ damage [2]. The most commonly observed types of diabetes are type 1 diabetes, type 2 diabetes, and gestational diabetes. Type 1 diabetes, also termed insulin-dependent diabetes (IDDM), is an autoimmune illness that usually occurs in childhood or adolescence and is marked by the devastation of insulin-producing beta cells in the pancreas. Type 2 diabetes, or non-insulin-dependent diabetes (NIDDM), is often connected to lifestyle issues like improper diet and lack of exercise. Gestational diabetes arises during pregnancy and usually resolves after delivery; however, it can elevate the likelihood of developing type 2 diabetes in the future [3].

In recent decades, the incidence of diabetes mellitus (DM) has risen dramatically. According to current reports, by 2045, over 629 million people aged 20-79 years will have diabetes [4]. Eighty percent of individuals with diabetes reside in low- and middle-income countries. In many of these regions, the occurrence of DM, once considered a rare condition in Africa, is increasing rapidly, with type 2 diabetes being the most common [5]. Initially, it was predicted that by 2025, the overall diabetes population would be in developing countries owing to factors like increasing life expectancy, an elderly population, and increased urbanization. Consequently, the long-term effects of diabetes will impact both individual and community health in these areas [6].

Genetics, older age, physical inactivity, inappropriate nutrition, and being overweight are among the risk factors that contribute to the progression of diabetes. Additional risk factors may include gestational diabetes, a past history of diabetes, and some illnesses like hypertension and raised cholesterol levels [7].

The severity of diabetes symptoms varies based on the type and duration of the condition. Some people, especially those with early-stage type 2 diabetes, may be asymptomatic, while others, particularly children with severe insulin deficiency and high hyperglycemia, may experience frequent urination, dehydration, increased appetite, loss of weight, and blurry vision. Without proper management, diabetes can lead to complications like ketoacidosis or, less commonly, non-ketotic hyperosmolar syndrome, potentially resulting in stupor, coma, and, if untreated, even death [8].

Skin issues are among the numerous complications of diabetes, affecting up to 80% of patients and greatly disturbing their quality of life. These complications also contribute to higher morbidity and mortality rates. Common diabetes-related skin conditions include diabetic dermopathy, skin infections, and xerosis [9].

Type 2 diabetes patients are more vulnerable to infections, with the urinary tract infection (UTIs) being the most common [10]. Factors that increase the risk of UTIs in diabetes are age, metabolic control, and long-standing complications, mainly diabetic nephropathy and cystopathy [11].

Patients with diabetes face noticeably higher rates of many wound complications, like infections, dehiscence, and scarring. They are also at a heightened risk for surgical site infections across most specialties, even when compared to cases with elevated post-operative glucose levels [12]. Moreover, comorbidities like obesity further increase the possibility of both superficial and deep incisional surgical site infections [13].

Nutritional status is an essential predictor of wound healing outcomes [14]. For patients with type 2 diabetes, it acts as a key independent indicator of infection risk and overall healing success in surgical wounds. Poor nutrition is closely associated with more severe wounds, as evaluated by subjective global assessment (SGA) and Wagner grading, a widely used scale to assess the severity of diabetic ulcers [15].

In Pakistan, there is insufficient data about how the duration of type 2 diabetes influences the progression of clinical characteristics and complications. Understanding these associations can guide healthcare professionals in emerging targeted strategies to report both the common and unique challenges faced by diabetic patients at different stages of their disease. Therefore, in this study, we evaluated the various symptoms of diabetes and their relation with the duration of diabetes. This can help the clinicians and endocrinologists to better assess the various symptoms of diabetes with respect to the duration of the disease.

## Materials and methods

This cross-sectional study was conducted in a secondary care hospital using a non-probability convenient sampling method. Patients visiting the outpatient clinics were recruited after taking their written informed consent. The study was ethically approved by the Ethical Review Board of the concerned hospital. The duration of the study was about six months March 1, 2024, to August 31, 2024. This study included 450 type 2 diabetic patients aged 40-65 years, categorized into three groups of 150 in each group: Group A consisted of patients with diabetes for less than one year, Group B consisted of patients with diabetes for one to five years, and Group C consisted of patients with diabetes more than five years. The study excluded individuals with type 1 diabetes, those with hypoglycemia, individuals who had undergone any surgical procedures, and those who had received chemotherapy.

Clinical assessments were conducted to evaluate the participants' glycemic control and potential complications associated with type 2 diabetes mellitus (T2DM). Glycemic control was measured using the following primary indicators: glycosylated hemoglobin (HbA1c) levels and postprandial glucose levels. The study identified patients with type 2 diabetes by using their latest HbA1c levels, which reflect glycemic control. Postprandial glucose levels were measured two hours after a standard meal, allowing for a comprehensive understanding of the participants' glucose metabolism. In addition to glycemic assessment, a thorough evaluation of diabetic complications was performed. Cardiovascular health was assessed through physical examinations, including blood pressure measurements and heart rate assessments, as well as laboratory tests to measure lipid profiles. Demographic data were collected via structured questionnaires, which included questions about age, sex, and lifestyle factors such as physical activity levels, dietary habits, and smoking status. Height and weight were evaluated to determine body mass index (BMI). Data on recent medical history and previous sleep issues - including insomnia, unusual behaviors during sleep, and difficulty sleeping at the desired time - were collected through a questionnaire. Symptoms suggesting dry eyes were identified on the basis of a history of ocular discomfort, such as tenderness, gritty sensation, irritation, inflammation, and blurred vision that got better with blinking and increased tear production. Additionally, heart rate and random blood sugar levels were measured.

Data was analyzed using IBM SSPS Statistics version 20.0 (IBM, Armonk, NY). The socio-demographic details and signs and symptoms related to type 2 diabetes were reported in frequencies and percentages. Quantitative variables were represented as means with standard deviations. A chi-square test was used to examine the association of clinical symptoms in type 2 diabetes mellitus. Additionally, an one-way ANOVA was employed to analyze the relationship between the means of demographic variables. A p-value < 0.05 was reflected as statistically significant.

## Results

This study involved 450 type 2 diabetic patients divided into three groups of 150 patients each. The mean age was significantly higher in patients with diabetes in group A as compared to group B and group C patients (p <0.001). Similarly, the mean weight was highest among patients in group A (72.59±13.30 kg) and lowest among patients in group B (66.54±15.21 kg), with patients in group C having a mean weight of 65.83±14.74 kg, with a statistically significant difference (p<0.001). Regarding height, patients with diabetes in group A had a mean height of 68.00±10.93 inches, while those with diabetes in group B had a mean height of 64.96±7.86 inches, and those in group C had a mean height of 68.44±11.35 inches, with a statistically significant difference among them (p=0.006). Heart rate was also notably different among the groups, showing significant differences (p<0.001). Finally, the mean random blood sugar (RBS) was highest in patients in group A (334.92±96.32 mg/dL) and lowest in patients in group C (227.63±89.04 mg/dL), with a significant difference observed among them (p<0.001), as presented in Table 1.

**Table 1:** The association of demographic information of the patients with type 2 diabetes with respect to duration of diabetes (n=450)

Variables	Duration of Diabetes				
	Group A, Mean±SD	Group B, Mean±SD	Group C, Mean±SD	f-value	p-value
Age (Years)	60.44±15.88	52.26±14.37	53.95±14.28	12.676	<0.001
Weight (kg)	72.59±13.304	66.54±15.21	65.83±14.74	9.918	<0.001
Height (inch)	68.00±10.93	64.96±7.86	68.44±11.35	5.180	0.006
Heart rate (beats/min)	87.97±10.30	80.64±11.36	86.56±11.45	18.594	<0.001
Random blood sugar (RBS) (mg/dL)	334.92±96.32	332.54±112.44	227.63±89.04	56.585	<0.001

The association of gender, comorbidities, and socioeconomic status with the duration of diabetes showed that there was a significant gender difference in the duration of diabetes (p<0.001). All participants (150) in group A were male patients (100.0%). However, in group B, only 36 (24.0%) were male patients and the remaining 114 (76.0%) were female patients. For patients with diabetes in group C, 83 (55.3%) were male patients and 67 (44.7%) were female patients. Similarly, socioeconomic status showed significant differences with the diabetes duration (p=0.002). A significant association was seen between dyslipidemia and the duration of diabetes (p<0.001). Dyslipidemia was most common in group B (126 patients, 84.0%) and least common in group C (88 patients, 58.7%). Smoking and depression were also significantly associated with the duration of diabetes (p<0.001), as presented in Table 2.

**Table 2:** The association of gender, comorbidities, and socioeconomic status with respect to duration of diabetes

Variables		Duration of Diabetes				
		Group A, n (%)	Group B, n (%)	Group C, n (%)	Pearson chi-Square	p-value
Gender	Male	150 (100.0%)	36(24.0%)	83(55.3%)	182.01	<0.001
	Female	0 (0.0%)	114(76.0%)	67(44.7%)		
Socioeconomic Status	Low	24 (16.0%)	31(20.7%)	23(15.3%)	16.67	0.002
	Middle	77 (51.3%)	86(57.3%)	105(70.0%)		
	High	49 (32.7%)	33(22.0%)	22(14.7%)		
Hypertension	Yes	104 (69.3%)	104 (69.3%)	98 (65.3%)	0.73	0.692
	No	46 (30.7%)	46 (30.7%)	52 (34.7%)		
Dyslipidemia	Yes	112 (74.7%)	126 (84.0%)	88 (58.7%)	24.66	<0.001
	No	38 (25.3%)	24 (16.0%)	62 (41.3%)		
Smoking	Yes	73 (48.7%)	21 (14.0%)	42 (28.0%)	43.26	<0.001
	No	77 (51.3%)	129 (86.0%)	108 (72.0%)		

The association of renal, ocular, and respiratory symptoms in patients with type 2 diabetes showed that frequent urination was significantly more common in group A (89 patients, 59.3%) compared to group B (54 patients, 36.0%) and group C (11 patients, 7.3%) (p<0.001). The frequency of nighttime urination also shows a similar pattern, with the highest occurrence in patients in group C, particularly those who urinate three times at night 125 (83.3%), and in group B, 71 patients (47.3%) urinate every two hours, with a significant relationship among them (p<0.001). Blurry vision was also significantly associated with the duration of diabetes (p=0.002). Edema was more frequently bilateral in group A 79 (52.7%), while unilateral edema was more common in those with diabetes in group C (108 patients, 72.0%) (p<0.001). Among those with bilateral edema, moderate edema was most common in group B (106 patients, 70.7%), whereas mild edema was more prevalent in group A diabetes (88 patients 58.7%) (p<0.001). Shortness of breath was reported by the majority of patients in group A (104 patients, 69.3%) and decreased significantly with the longer duration of diabetes (p=0.012). Difficulty in breathing showed a significant variation among all groups of patients with diabetes (p<0.001). Chest tightness or pressure was most common in group B patients compared to group A and group C patients, with a significant association among them (p<0.001), as presented in Table 3.

**Table 3:** The association of renal, ocular, and respiratory symptoms in type 2 diabetes patients with respect to duration of diabetes

Variables		Duration of Diabetes				
		Group A, n (%)	Group B, n (%)	Group C, n (%)	Pearson chi-Square	p-value
Frequent urination	Yes	89 (59.3%)	54 (36.0%)	11 (7.3%)	90.40	<0.001
	No	61 (40.7%)	96 (64.0%)	139 (92.7%)		
Urination at night	Three times at night	71 (47.3%)	91 (60.7%)	125 (83.3%)	44.84	<0.001
	Every two hours	71 (47.3%)	52 (34.7%)	25 (16.7%)		
	Every hour	8 (5.3%)	7 (4.7%)	0 (0.0%)		
Poor night vision	Yes	54 (36.0%)	54 (36.0%)	41 (27.3%)	3.39	0.183
	No	96 (64.0%)	96 (64.0%)	109 (72.7%)		
Blurry vision	Yes	41 (27.3%)	70 (46.7%)	55 (36.7%)	12.04	0.002
	No	109 (72.7%)	80 (53.3%)	95 (63.3%)		
Edema if yes then	Bilateral	72 (48.0%)	79 (52.7%)	42 (28.0%)	21.03	<0.001
	Unilateral	78 (52.0%)	71 (47.3%)	108 (72.0%)		
If bilateral then	1+ Mild (Both ankles/feet)	88 (58.7%)	26 (17.3%)	57 (38.0%)	61.18	<0.001
	2+ Moderate (Both feet, hands, lower arms and lower legs)	50(33.3%)	106 (70.7%)	67 (44.7%)		
	3+ Severe (Generalized bilateral pitting edema)	12 (8.0%)	18 (12.0%)	26 (17.3%)		
Confusion	Yes	66 (44.0%)	59 (39.3%)	43 (28.7%)	7.92	0.019
	No	84 (56.0%)	91 (60.7%)	107 (71.3%)		
Shortness of breath	Yes	104 (69.3%)	88 (58.7%)	79 (52.7%)	8.92	0.012
	No	46 (30.7%)	62 (41.3%)	71 (47.3%)		
Dyspnea grading	While climbing stairs	80 (53.3%)	75 (50.0%)	68 (45.3%)	16.78	0.010
	While walking for more than 6 hours in a day	42 (28.0%)	63 (42.0%)	59 (39.3%)		
	While walking for less than 6 hours in a day"	16 (10.7%)	9 (6.0%)	8 (5.3%)		
	While at rest	12 (8.0%)	3 (2.0%)	15 (10.0%)		
Difficulty in breathing If yes	Mild	44 (29.3%)	89 (59.3%)	41 (27.3%)	43.03	<0.001
	Moderate	80 (53.3%)	47(31.3%)	74(49.3%)		
	Severe	26 (17.3%)	14 (9.3%)	35 (23.3%)		
Chest tightness or pressure	Yes	104 (69.3%)	121 (80.7%)	77 (51.3%)	29.74	<0.001
	No	46 (30.7%)	29 (19.3%)	73 (48.7%)		

The association of psychological and gastrointestinal symptoms in patients with type 2 diabetes showed that tingling or numbness in the hands or feet was significantly more common in patients in group C (121 patients, 80.7%) compared to those in group A (92 patients, 61.3%) and group B (74 patients, 49.3%) (p<0.001). Burning discomfort in the legs or feet was most prevalent in patients in group B (107 patients, 71.3%) and was significantly associated with the duration of diabetes (p<0.001). Sensitivity in the feet was more common in patients in group B as compared to the other groups (p=0.003). Muscular pain or cramps in the legs or feet were significantly more prevalent in patients in groups A and C (p<0.001). Loss of appetite and insomnia showed no significant differences across the groups. Increased thirst was notably more common in patients in group B compared to patients in other groups (p<0.001), while fatigue was highly prevalent across all groups but significantly greater in patients in group B (p<0.001). Psychologically, irritability or mood changes were most prevalent in patients in group B and significantly related to the duration of diabetes (p<0.001). Other psychological symptoms such as feeling tired and weak were also significantly associated with the duration of diabetes (p<0.001), with varying prevalence across the groups, as presented in Table 4.

**Table 4:** The association of psychological and gastrointestinal symptoms in type 2 diabetes patients with respect to duration of diabetes

Variables		Duration of Diabetes				
		Group A, n (%)	Group B, n (%)	Group C, n (%)	Pearson chi-Square	p-value
Tingling or numbness in the hands or feet	Yes	92 (61.3%)	74 (49.3%)	121 (80.7%)	32.45	<0.001
	No	58 (38.7%)	76 (50.7%)	29 (19.3%)		
Burning pain in legs or feet	Yes	81 (54.0%)	107 (71.3%)	70 (46.7%)	19.67	<0.001
	No	69 (46.0%)	43 (28.7%)	80 (53.3%)		
Too sensitive feet on touch	Yes	43 (28.7%)	57 (38.0%)	30 (20.0%)	11.83	0.003
	No	107 (71.3%)	93 (62.0%)	120 (80.0%)		
Muscular pain or cramps in legs	Yes	125 (83.3%)	144 (96.0%)	143 (95.3%)	19.71	<0.001
	No	25 (16.7%)	6 (4.0%)	7 (4.7%)		
Loss of appetite	Yes	94 (62.7%)	88 (58.7%)	83 (55.3%)	1.67	0.434
	No	56 (37.3%)	62 (41.3%)	67 (44.7%)		
Insomnia	Yes	76 (50.7%)	66 (44.0%)	66 (44.0%)	1.78	0.409
	No	74 (49.3%)	84 (56.0%)	84 (56.0%)		
Increased thirst	Yes	57 (38.0%)	95 (63.3%)	49 (32.7%)	32.58	<0.001
	No	93 (62.0%)	55 (36.7%)	101 (67.3%)		
Fatigue	Yes	126 (84.0%)	143 (95.3%)	118 (78.7%)	18.05	<0.001
	No	24 (16.0%)	7 (4.7%)	32 (21.3%)		
Increased hunger	Yes	38 (25.3%)	45 (30.0%)	30 (20.0%)	3.99	0.136
	No	112 (74.7%)	105 (70.0%)	120 (80.0%)		
Feeling tired and weak	Yes	105 (70.0%)	129 (86.0%)	103 (68.7%)	14.84	0.001
	No	45 (30.0%)	21 (14.0%)	47(31.3%)		
Mood changes	Yes	106 (70.7%)	133 (88.7%)	110 (73.3%)	16.26	<0.001
	No	44 (29.3%)	17 (11.3%)	40 (26.7%)		

## Discussion

The incidence of type 2 DM is increasing, along with its complications, such as coronary heart disease (CHD) [16]. Therefore, this study demonstrated clinical manifestations in type 2 DM patients based on the duration of their diabetes.

A cross-sectional research involving 590 T2DM patients explored the relationship between diabetes-related symptoms and gender. Among these patients, 310(52.5%) were male, and 280 (47.5%) were female. The mean ages of male and female patients were 57.46±14.93 and 50.38±14.85 years, correspondingly, showing a significant association between genders (p<0.001). The study found a significant association between renal manifestations and gender in type 2 diabetes patients (p<0.05). Ocular manifestations, including distortion and blurred vision, had also significant relationship with gender (p<0.05). Additionally, a significant relationship was observed between gender and respiratory manifestations, like shortness of breath, dyspnea severity, and chest pain severity (p <0.05) [17]. In the present study, the mean age was notably higher in group A patients with diabetes (60.44±15.88 years) compared to those with diabetes in group B (52.26±14.37 years) and group C (53.95±14.28 years) (p<0.001). The present study also revealed significant associations between the duration of diabetes and renal manifestations, particularly in patients with shorter durations of diabetes (p <0.001). Furthermore, significant relationships were observed between the duration of diabetes and ocular and respiratory manifestations, with higher rates of blurry vision, edema, and respiratory issues in group A, while these symptoms tended to decrease in prevalence as the duration of diabetes increased.

Similarly, another study found that the average age of diabetes patients at presentation was 50±11 years. The majority of these patients (37%) had been living with diabetes for 10 years or more, with the mean duration being 8.5 years [18]. This aligns with the results of Ahmed et al., who stated a mean age of 54 years for patients with diabetes [19]. Basit et al. also observed a similar mean age at presentation, noting that most patients had long-standing diabetes, which significantly impacted their social and professional lives [20]. In contrast, the present study differed from these previous findings. It categorized 450 patients into three groups - 150 in each group - based on their diabetes duration: less than one year, one to five years, and more than five years. Moreover, the present study revealed that the mean age was significantly higher in patients with diabetes in group A (60.44±15.88 years) compared to those with patients with diabetes in group B (52.26±14.37 years) and group C (53.95±14.28 years) (p<0.001).

Likewise, another study found a slight predominance of male patients (52.5%) compared to female patients with diabetes (47.5%) [17]. These findings align with the current study, which indicated that all participants in group A were male patients (150, 100%). However, in the group B, only 36 (24.0%) were male patients, while the remaining 114 (76.0%) were female patients. Among patients with diabetes in group C, 83 (55.3%) were male patients and 67 (44.7%) were female patients.

Diabetes is a common reason for nocturia for several reasons. Hyperglycemia-induced osmotic diuresis can significantly increase urine production during the night [21]. A meta-analysis explored the relationship between diabetes and nocturia, finding that diabetes nearly doubled the occurrence of nocturia among the 197,809 diabetes patients assessed. The prevalence of nocturia increase in both male (p<0.001) and female (p <0.001) patients with diabetes, with a stronger correlation observed in men [22]. The present study also identified significant links between the diabetes duration and renal symptoms, showing that patients with shorter diabetes durations experienced more frequent urination and nighttime urination (p<0.001).

This study had a few limitations. The cross-sectional design limits the ability to establish causal relationships. Relying on self-reported data may introduce observer and recall bias, and focusing on a single population could restrict the generalizability of the findings. Future research should involve longitudinal studies to monitor disease progression over time and involve a more diverse population to enhance the relevance of the results. Additionally, incorporating objective measures alongside self-reported data could improve the accuracy of the findings.

## Conclusions

Diabetes mellitus, especially type 2 diabetes is a very prevalent systemic disease, with various musculoskeletal and psychosocial symptoms. The frequency of these symptoms varies with respect to the duration of diabetes. In this study, we outlined various symptoms of diabetes and their relation to the duration of diabetes. Among various symptoms, dyspnea, chest tightness, and muscle cramps increase with the increasing duration of diabetes. Conversely, some of the symptoms such as frequent urination are more common in the early stages of the disease. It is important for clinicians to thoroughly investigate the various symptoms of diabetes as these symptoms vary with respect to the duration of the disease.
